# Supplementary material for: A Clinical Probability-Based, Stepwise Algorithm for the Diagnosis of Giant Cell Arteritis: Study Protocol and Baseline Characteristics of the First 50 Patients Included in the Prospective Validation Study with Focus on Cranial Symptoms
Source: J Clin Med. 2025 Mar 26;14(7):2254. doi: 10.3390/jcm14072254 (PMC11989727; doi:10.3390/jcm14072254)
Supplement: Supplementary file 1 [file jcm-14-02254-s001.zip › jcm-3500406-supplementary.pdf]

Journal of Clinical Medicine

MDPI

Patient questionnaire (Version 1.4, English translation):

Date of examination: DD.MM.YYYY

Demographic data

1.0 Gender:

- ○ male
- ○ female

1.1 Date of birth:

____

1.2 Age in years:

- ○ <65 years
- ○ 65-74 years
- ○ ≥75 years

1.3 Age for clinical probability of GCA:

- ○ <70 years
- ○ >/= 70 years

1.4 Body height (in meters):

____

1.5 Body weight (in kg):

____

Ophthalmologic symptoms/findings

1.0 Is a visual impairment present?:

- ○ yes
- ○ no
- ○ unknown

1.1 Which eye is affected:

- ○ right
- ○ left
- ○ bilateral

1.2 Date of onset of visual impairment:

____

2.0 Visual acuity left:

____

2.1 Visual acuity right:

____

Symptoms

1.0 New-onset headache:

- ○ yes
- ○ no
- ○ unknown

I. Please take a headache anamnesis if headaches are present

I.a) Have you suffered from headaches before?:

- ○ yes
- ○ no
- ○ unknown

I.b) Pain present daily:

- ○ yes
- ○ no
- ○ unknown

I.c) Headache currently present?:

- ☐ yes
- ☐ no
- ☐ unknown

I.d) Duration of the new headache:

- ○ <7 days
- ○ 7-14 days
- ○ 14-28 days
- ○ >28 days

I.e) At what time of the day does the pain peak?:

- ☐ in the morning
- ☐ at noon
- ☐ in the evening
- ☐ at night

II. Localization of pain

II.a):	II.b) (multiple choice):	II.c) Pain at the temporal artery:
○ unilateral	☐ eyes	○ yes
○ bilateral	☐ face	○ no
	☐ back of the head	○ unknown
	☐ neck	
	☐ ears	
	☐ vertex	
	☐ temples	
	☐ forehead	

III. Pain characteristics

III.a) Description of pain quality	III.b) Pain onset:	III.c) Daily course of pain: (multiple choice):
☐ stabbing	○ sudden	☐ undulating
☐ pulsating	○ within a short time	☐ permanent-increasing
☐ throbbing	○ gradually	☐ permanent-decreasing
☐ dull		☐ permanent-stable
☐ hammering		☐ paroxysmal
☐ sore-like		
☐ squeezing		
☐ drilling		
☐ constrictive		

IV. Headache intensity

IV.a) Please determine the intensity of pain using the shown scale:

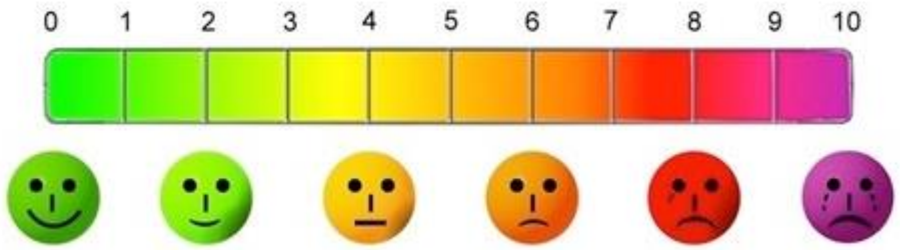

No pain | Mild | Moderate | Strong | Severe | Strongest pain imaginable

V. Quality of life

V.a) Please determine the impairment in activities of daily life using the shown scale:

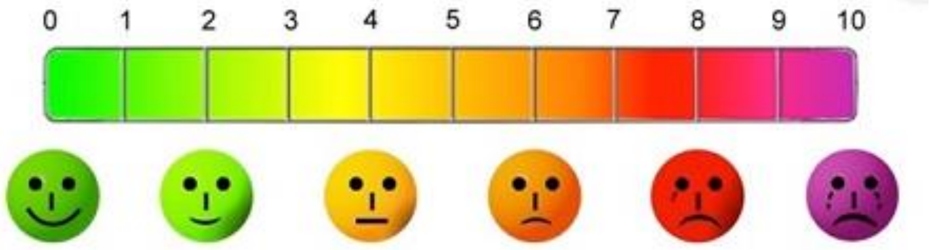

No impairment | Mild impairment | Medium impairment | Strong impairment | Very strong impairment | Incapable of participating in activities of daily life

1.2 Jaw claudication:

- ○ yes
- ○ no
- ○ unknown

1.3 Combing pain / scalp tenderness:

- ○ yes
- ○ no
- ○ unknown

2.0 Diplopia:

- ○ yes
- ○ no
- ○ unknown

2.1 If yes, on which side:

- ○ right
- ○ left
- ○ bilateral

3.0 Amaurosis fugax:
- yes
- no
- unknown

3.1 If yes, on which side:
- right
- left
- bilateral

4.0 Persistent reduction or loss of vision:
- yes
- no
- unknown

4.1 If yes, on which side:
- right
- left
- bilateral

4.2 When was the visual impairment or loss noticed?
- after night sleep
- after hemodialysis
- after surgery
- no statement possible

4.3 Please specify date and time:

____

5.0 Polymyalgia rheumatica:
- yes
- no
- unknown

6.0 Fever (≥ 38 °C) in the last 14 days:
- yes
- no
- unknown

7.0 Night sweats (in the last 7 days):

- ○ yes
- ○ no
- ○ unknown

8.0 Limb claudications:

- ○ yes
- ○ no
- ○ unknown

8.1. If yes, on which side (multiple selections possible):

____

9.0. AION:	9.1. Snoring:	9.2 Daytime sleepiness:
○ yes	○ yes	○ yes
○ no	○ no	○ no
○ unknown	○ unknown	○ unknown

VI. In case of daytime sleepiness, please complete the OSAS questionnaire

VI.a) Do you snore loudly (to an extent that it is audible through closed doors or that your partner nudges you by elbow at night due to your snoring)?:

- ○ yes
- ○ no
- ○ unknown

VI.b) Are you often tired, exhausted or sleepy during the day (e.g. while driving)?:

- ○ yes
- ○ no
- ○ unknown

VI.c) Has anyone ever noticed that you stop breathing or cannot breathe/gasp for air during sleep?

- yes
- no
- unknown

VI.d) Body Mass Index over 35 kg/m2:

- yes
- no
- unknown

VI.e) Are you over 50 years old?:

- yes
- no
- unknown

VI.f) Please measure the neck circumference around the throat. Is the value ≥43 cm (men) or ≥41 cm (women)?:

- yes
- no
- unknown

**Pre-existing conditions:**	
1.0 Vascular disease:<br>○ yes<br>○ no<br>○ unknown	1.1 Which vascular diseases are present?:<br><br>____________________________________
2.0 Atrial fibrillation:<br>○ yes<br>○ no<br>○ unknown	
3.0 Heart failure:<br>○ yes<br>○ no<br>○ unknown	
4.0 Stroke/TIA:<br>○ yes<br>○ no<br>○ unknown	
5.0 Arterial hypertension:<br>○ yes<br>○ no<br>○ unknown	
6.0 Diabetes mellitus:<br>○ yes<br>○ no<br>○ unknown	

7.0 Dyslipidemia:

- yes
- no
- unknown

8.0 Tobacco smoking:

- yes
- no
- stopped

9.0 OSAS:

- yes
- no
- unknown

10.0 History of cancer:

- yes
- no
- unknown

11.0 Known rheumatic disease:

- yes
- no
- unknown

**Current medication**	
1.0 Antiplatelets :	1.1 Please select:
◯ yes	☐ Aspirin
◯ no	☐ Clopidogrel
◯ unknown	☐ Prasugrel
	☐ Ticagrelor
2.0 Anticoagulation:	2.1 Please select:
◯ yes	☐ DOACs
◯ no	☐ Warfarins
◯ unknown	☐ Unknown
3.0 Lipid lowering medication:	3.1 Please select:
◯ yes	☐ Bempedoic acid
◯ no	☐ Ezitimibe
◯ unknown	☐ Inclisiran
	☐ PCSK9-Inhibitors
	☐ Statins
4.0 Glukocorticoid treatment:	4.1 Daily dose of prednisolon [mg]:
◯ yes	________________________________
◯ no	

◯ unknown	4.2 Start of glucocorticoid treatment:
	_________________________________
5.0 Immunosuppressants:	
◯ yes	
◯ no	
◯ unknown	

Physical examination

1.0 Temporal artery tenderness on palpation:

- ○ yes
- ○ no
- ○ unknown

2.0 Temporal artery induration:

- ○ yes
- ○ no
- ○ unknown

3.0 Temporal artery swelling:

- ○ yes
- ○ no
- ○ unknown

4.0 Bruit over the axillary artery:

- ○ yes
- ○ no
- ○ unknown
